# Supplementary material for: Two Glu/Asp residues cooperatively mediate an early step of ATP hydrolysis in GHKL ATPases MutL and GyrB
Source: eLife. 2026 Sep 25;15:RP111443. doi: 10.7554/eLife.111443 (PMC13614785; doi:10.7554/eLife.111443)
Supplement: Supplementary file 2 [file elife-111443-supp1.docx]

# Supplementary File 1. Primers used for site-directed mutagenesis in this study

| Target Protein | Mutation | Primer Name | Sequence (5′–3′) |
| --- | --- | --- | --- |
| aqMutL NTD | E32K | aqML_E32K_fw | ACTCGTTAAGAATTCTCTGGACGCAAA |
|  |  | aqML_E32K_rv | GAATTCTTAACGAGTTCCTTTACGAC |
|  | E32A | aqML_E32A_fw | CTCGTTGCGAATTCTCTGGACGCAAA |
|  |  | aqML_E32A_rv | AGAATTCGCAACGAGTTCCTTTACGA |
|  | E32V | aqML_E32V_fw | CTCGTTGTGAATTCTCTGGACGCAAA |
|  |  | aqML_E32V_rv | AGAATTCACAACGAGTTCCTTTACGA |
|  | E32G | aqML_E32G_fw | CTCGTTGGGAATTCTCTGGACGCAAA |
|  |  | aqML_E32G_rv | AGAATTCCCAACGAGTTCCTTTACGA |
|  | E32Q | aqML_E32Q_fw | CTCGTTCAGAATTCTCTGGACGCAAA |
|  |  | aqML_E32Q_rv | AGAATTCTGAACGAGTTCCTTTACGA |
|  | E29G | aqML_E29G_fw | GTAAAGGGACTCGTTGAGAATTCTCT |
|  |  | aqML_E29G_rv | AACGAGTCCCTTTACGACGTCAACAG |
|  | E29K | aqML_E29K_fw | GTAAAGAAACTCGTTGAGAATTCTCT |
|  |  | aqML_E29K_rv | AACGAGTTTCTTTACGACGTCAACAG |
|  | E29Q | aqML_E29Q_fw | CGTAAAGCAACTCGTTGAGAATTCTC |
|  |  | aqML_E29Q_rv | ACGAGTTGCTTTACGACGTCAACAGG |
|  | E29V | aqML_E29V_fw | GTAAAGGTACTCGTTGAGAATTCTCT |
|  |  | aqML_E29V_rv | AACGAGTACCTTTACGACGTCAACAG |
|  | E29Q/E32Q | aqML_E29Q/E32Q_fw | CGTAAAGCAACTCGTTCAGAATTCTC |
|  |  | aqML_E29Q/E32Q_rv | ACGAGTTGCTTTACGACGTCAACAGG |
| aqGyrB NTD | D51A | aqGyrB_D51A_fw | ATACTTGCCAACGCCGTAGATGAGGC |
|  |  | aqGyrB_D51A_rv | GGCGTTGGCAAGTATTTCCCAGATGA |
|  | D51N | aqGyrB_D51N_fw | AATACTTAACAACGCCGTAGATGAGG |
|  |  | aqGyrB_D51N_rv | GCGTTGTTAAGTATTTCCCAGATGAG |
|  | E48A | aqGyrB_E48A_fw | ATCTGGGCAATACTTGACAACGCCGT |
|  |  | aqGyrB_E48A_rv | AAGTATTGCCCAGATGAGGTGGTGAA |
|  | E48Q | aqGyrB_E48Q_fw | ATCTGGCAAATACTTGACAACGCCGT |
|  |  | aqGyrB_E48Q_rv | AAGTATTTGCCAGATGAGGTGGTGAA |
|  | E48Q/D51N | aqGyrB_E48Q/D51N_fw | AATACTTAACAACGCCGTAGATGAGG |
|  |  | aqGyrB_E48Q/D51N_rv | GCGTTGTtAAGTATTTGCCAGATGAG |
| human PMS2 NTD | E44Q | hPMS2_E44Q_fw | CCAGATGAAAGGCAAATTTTGCTAC |
|  |  | hPMS2_E44Q_rv | TGCCTTTCATCTGGAGTAACATTGAT |
|  | E44G | hPMS2_E44G_fw | TTAGTAGGAAACAGTCTGGATGCTGG |
|  |  | hPMS2_E44G_rv | ACTGTTTCCTACTAACTCCTTTACCG |
|  | E44V | hPMS2_E44V_fw | TTAGTAGTAAACAGTCTGGATGCTGG |
|  |  | hPMS2_E44V_rv | ACTGTTTACTACTAACTCCTTTACCG |
| human MLH1 NTD | E37G | hMLH1_E37G_fw | ATGATTGGGAACTGTTTAGATGCAAA |
|  |  | hMLH1_E37G_rv | ACAGTTCCCAATCATCTCTTTGATAG |
|  | E37K | hMLH1_E37K_fw | GATGATTAAGAACTGTTTAGATGCAA |
|  |  | hMLH1_E37K_rv | CAGTTCTTAATCATCTCTTTGATAGC |
|  | E37Q | hMLH1_E37Q_fw | GATGATTCAGAACTGTTTAGATGCAA |
|  |  | hMLH1_E37Q_rv | CAGTTCTGAATCATCTCTTTGATAGC |
