# Supplementary material for: Two Glu/Asp residues cooperatively mediate an early step of ATP hydrolysis in GHKL ATPases MutL and GyrB
Source: eLife. 2026 Sep 25;15:RP111443. doi: 10.7554/eLife.111443 (PMC13614785; doi:10.7554/eLife.111443)
Supplement: Peer review history

# Editor report: eLife Assessment

**DOI:** 10.7554/eLife.111443.4.sa0

**Authors:** Axel T Brunger

**Affiliations:** Stanford University School of Medicine, Howard Hughes Medical Institute United States

**Keywords:** Solid, Valuable

In this **solid** work, Fukui et al. re-examined the ATP hydrolysis mechanism in GHKL ATPases, revealing a cooperative role for two conserved acidic residues rather than a single one. This **valuable** study used a range of biochemical and structural techniques on various mutants from different members of the GHKL ATPase family to test and validate their proposed mechanism. An updated and extended mechanistic model of ATP hydrolysis by this class of enzymes is proposed.

# Referee report: Reviewer #1 (Public review)

**DOI:** 10.7554/eLife.111443.4.sa1

[Editors' note: this version has been assessed by the Reviewing Editor without further input from the original reviewers. We thank the authors for revising the manuscript according to the reviewers' comments. We have no further comments.]

In this manuscript the applicants study two residues in the GHKL ATPase active site of Aq MutL and GyrB, and argue that the catalytic base function is shared between two conserved acidic residues that are 3 residues apart.

In the manuscript, the authors generated mutant versions in MutL and GyrB (both ala and the appropriate Asn/Gln version) and performed ATPase analysis. They also generated high resolution crystal structures of the GyrB NTD with AMPPnP for WT and mutants of the two acidic residues. The data show that mutation in either of these residues does not fully kill activity (with the exception of the Alanine mutation of the first of the two, that interferes with ATP (or AMPPnP) binding). When the acidic residues are mutated to Asn/Gln, the catalytic water can still be positioned, and hence these mutants are more active than the Ala mutants. In both cases the double mutation is catalytic dead.

The authors then perform phylogenetic analysis and ancestral gene reconstruction and based on this they argue that HSP90 forms a different class of GHKL ATPases, and lost rather than gained this separate status.

# Referee report: Reviewer #2 (Public review)

**DOI:** 10.7554/eLife.111443.4.sa2

Summary:

In this manuscript, Fukui et al. re-examined the ATP hydrolysis mechanism in GHKL ATPases, revealing a cooperative role of two conserved acidic residues rather than one. The authors have used a range of biochemical and structural techniques on various mutants from different members of the GHKL ATPase family to test and validate their proposed mechanism.

Through a detailed re-analysis of their previously published structure of the aqMutL NTD (ATPase domain) in complex with AMPPCP, they identified Glu29 and Glu32 as interacting with nucleophilic water for the catalysis. The authors carefully dissected the respective roles of these two acidic residues with a series of site-directed mutations. Mutations at Glu29 impaired ATPase activity without affecting protein secondary structure or ATP binding in the case of the E29Q mutant. Moreover, mutations at Glu32 did not affect secondary structure (except for E32G) but reduce ATPase activity. Activity was abolished when both residues (E29Q/E32Q) are mutated.

The authors extended their study to another GHKL ATPase, aqGyrB. Their findings further supported the cooperative function of the corresponding acidic residues in aqGyrB (Glu48 and Asp51) during ATP hydrolysis. Mutation of these residues partially impaired ATP hydrolysis without affecting protein secondary structure. ATPase activity was completely lost in the double mutant E48Q/D51M. While the E48Q mutant retained the ability to bind ATP, the E48A mutant did not. High-resolution structures of the WT and E48A, E48Q, D51A and D51N mutants of the aqGyrB NTD demonstrated that nucleophilic water positioning depended on these residues. E48 played a dominant role in water positioning and is critical for stabilising ATP lid formation and associated conformational changes, whereas D51 contributed cooperatively to catalysis.

The authors investigated the functional impact of mutating the corresponding residues in the human MutL homologs PMS2 and MLH1. Clinical variants consistently exhibited reduced or abolished ATPase activity, providing a potential molecular basis for Lynch syndrome, through impaired DNA mismatch repair.

Lastly, through evolutionary analysis, the authors inferred that the second acidic residue was likely present in the common ancestor of MutL, GyrB, and MORC proteins, but was lost in the case of Hsp90.

Strengths:

(1) This study contains a detailed structural and biochemical analysis of a biologically important set of GHKL ATPases. The authors identify a second acidic residue that is conserved and contributes to catalysis in a large subset of GHKL ATPases. An updated and extended mechanistic model of ATP hydrolysis by this class of enzymes is proposed, which involves cooperative and partially overlapping roles for the catalytic residue pair. This revised mechanistic model is invaluable for the interpretation of clinical variants of GHKL ATPases such as PMS2 and MLH1.

(2) The work described was performed to an excellent and rigorous technical standard. The structural and biochemical data are sound. The evidence supporting the claims is compelling.

Weaknesses:

(1) The identification in this study of a second acidic residue contributing to catalysis but not absolutely essential for catalysis is a useful finding. However, given that many structures of GHLK ATPases have been determined with different nucleotide analogs bound and that the essential role of the first acidic residue is well established, the importance and scope of the advances described here remain focused within the field of study of GHKL ATPases.

(2) The authors assessed the consequences of variants in the human MutL homologs PMS2 and MLH1, but various other human GHKL ATPases contain clinically relevant variants, some of which have stronger disease associations than the mutations examined in this study. A broader analysis of any effect of disease-linked mutations in GHKL ATPases would have strengthened this study.

(3) The effect of other aqMutL NTD E32 mutants, particularly, the E32K mutant on ATP binding remains unclear, although experimental assessment of nucleotide binding would be challenging due to the high protein concentrations required for the equilibrium dialysis assay.

# Author comment: Author response

**DOI:** 10.7554/eLife.111443.4.sa3

**Authors:** Kenji Fukui, Ayaka Shibuya, Takeshi Murakawa, Takato Yano

**Affiliations:** Nara Womens University Nara Japan; Osaka Medical and Pharmaceutical University Takatsuki Japan; Osaka Medical and Pharmaceutical University Takatsuki Japan; Osaka Medical and Pharmaceutical University Takatsuki Japan

The following is the authors’ response to the previous reviews.

**Public Reviews:**

**Reviewer #1 (Public review):**

Summary:

In this manuscript the applicants study two residues in the GHKL ATPase active site of Aq MutL and GyrB, and argue that the catalytic base function is shared between two conserved acidic residues that are 3 residues apart.

In the manuscript, they generated mutant versions in MutL and GyrB (both ala and the appropriate Asn/Gln version) and performed ATPase analysis. They also generated high resolution crystal structures of the GyrB NTD with AMPPnP for WT and mutants of the two acidic residues. The data show that mutation in either of these residues does not fully kill activity (with the exception of the Alanine mutation of the first of the two, that interferes with ATP (or AMPPnP) binding). When the acidic residues are mutated to Asn/Gln, the catalytic water can still be positioned, and hence these mutants are more active than the Ala mutants. In both cases the double mutation is catalytic dead.

The authors then perform phylogenetic analysis and ancestral gene reconstruction and based on this they argue that HSP90 forms a different class of GHKL ATPases, and lost rather than gained this separate status.

Strengths:

The biochemical analysis seems solid.

Weaknesses:

A major question that remains, is why the mutations have so much more detrimental effect in MutL (100-fold lower kcat/KM) than they do in GyrB (3-fold lower). Can the authors explain this? Doesn't this argue against the proposed catalytic conservation?

The authors need to discuss this issue explicitly to make it clear that conservation of the mechanism is not complete and that other interpretations are possible.

The structure figures all have omit maps for just the AMPPnP and the water, whereas the density for the the acidic residues and their mutants are not shown.

This has been addressed.

There are some issues with figure S2B and S5.

**Reviewer #2 (Public review):**

Summary:

In this manuscript, Fukui et al. re-examined the ATP hydrolysis mechanism in GHKL ATPases, revealing a cooperative role of two conserved acidic residues rather than one. The authors have used a range of biochemical and structural techniques on various mutants from different members of the GHKL ATPase family to test and validate their proposed mechanism.

Through a detailed re-analysis of their previously published structure of the aqMutL NTD (ATPase domain) in complex with AMPPCP, they identified Glu29 and Glu32 as interacting with nucleophilic water for the catalysis. The authors carefully dissected the respective roles of these two acidic residues with a series of site-directed mutations. Mutations at Glu29 impaired ATPase activity without affecting protein secondary structure or ATP binding in the case of the E29Q mutant. Moreover, mutations at Glu32 did not affect secondary structure (except for E32G) but reduce ATPase activity. Activity was abolished when both residues (E29Q/E32Q) are mutated.

The authors extended their study to another GHKL ATPase, aqGyrB. Their findings further supported the cooperative function of the corresponding acidic residues in aqGyrB (Glu48 and Asp51) during ATP hydrolysis. Mutation of these residues partially impaired ATP hydrolysis without affecting protein secondary structure. ATPase activity was completely lost in the double mutant E48Q/D51M. While the E48Q mutant retained the ability to bind ATP, the E48A mutant did not. High-resolution structures of the WT and E48A, E48Q, D51A and D51N mutants of the aqGyrB NTD demonstrated that nucleophilic water positioning depended on these residues. E48 played a dominant role in water positioning and is critical for stabilising ATP lid formation and associated conformational changes, whereas D51 contributed cooperatively to catalysis.

The authors investigated the functional impact of mutating the corresponding residues in the human MutL homologs PMS2 and MLH1. Clinical variants consistently exhibited reduced or abolished ATPase activity, providing a potential molecular basis for Lynch syndrome, through impaired DNA mismatch repair.

Lastly, through evolutionary analysis, the authors inferred that the second acidic residue was likely present in the common ancestor of MutL, GyrB, and MORC proteins, but was lost in the case of Hsp90.

Strengths:

(1) This study contains a detailed structural and biochemical analysis of a biologically important set of GHKL ATPases. The authors identify a second acidic residue that is conserved and contributes to catalysis in a large subset of GHKL ATPases. An updated and extended mechanistic model of ATP hydrolysis by this class of enzymes is proposed, which involves cooperative and partially overlapping roles for the catalytic residue pair. This revised mechanistic model is invaluable for the interpretation of clinical variants of GHKL ATPases such as PMS2 and MLH1.

(2) The work described was performed to an excellent and rigorous technical standard. The structural and biochemical data are sound. The evidence supporting the claims is compelling.

Weaknesses:

(1) The identification in this study of a second acidic residue contributing to catalysis but not absolutely essential for catalysis is a useful finding. However, given that many structures of GHLK ATPases have been determined with different nucleotide analogs bound and that the essential role of the first acidic residue is well established, the importance and scope of the advances described here remain focused within the field of study of GHKL ATPases.

(2) The authors assessed the consequences of variants in the human MutL homologs PMS2 and MLH1, but various other human GHKL ATPases contain clinically relevant variants, some of which have stronger disease associations than the mutations examined in this study. A broader analysis of any effect of disease-linked mutations in GHKL ATPases would have strengthened this study.

(3) The effect of other aqMutL NTD E32 mutants, particularly, the E32K mutant on ATP binding remains unclear, although experimental assessment of nucleotide binding would be challenging due to the high protein concentrations required for the equilibrium dialysis assay.

We are grateful to the Editors and reviewers for their careful assessment of our revised manuscript and for identifying the remaining points that required clarification. We have addressed each of these comments in the present revision. We believe that these revisions have resolved the remaining concerns and have further improved the clarity and accuracy of the manuscript.

**Recommendations for the authors:**

**Reviewer #1 (Recommendations for the authors):**

(1) Please discuss the large difference in the effect of the mutants on activity explicitly

According to the reviewer’s suggestion, we have added the following discussion to the revised manuscript:

“Although mutation of the two acidic residues impaired the ATPase activity in both aqMutL and aqGyrB, the magnitude of the effects differed substantially, with much greater reduction in the catalytic efficiency in aqMutL than in aqGyrB (Table 1). The molecular basis for this quantitative difference is currently unclear. One possible explanation is that subtle differences in the active-site architecture and surrounding residues alter the relative contribution of each acidic residue to catalysis, allowing aqGyrB to tolerate perturbation of either residue more effectively than aqMutL.” (p.6 line 257-262 in the revised manuscript)

(2) Figure S2B is a completely different view from the other panels, please provide the correct one.

We have revised Supplementary Fig. S2B so that the E48A structure is now shown from a viewpoint as similar as possible to those used in the other panels. We note, however, that the E48A structure cannot appear completely identical to the other panels because the E48A mutant does not bind the ATP analog and therefore does not undergo the nucleotide-binding-associated conformational changes observed in the other structures.

(3) S5 : It is not clear to me what is meant by " The scale bar indicates the number of amino acid substitutions per site." : there is a 'Tree Scale 1" but no other numbers in my version.

We thank the reviewer for pointing out that the scale bar in Supplementary Fig. S5 was insufficiently explained. The value “1” in the tree scale corresponds to a branch length of one amino acid substitution per site. To avoid ambiguity, we have revised the scale-bar label in Supplementary Fig. S5 to explicitly indicate “1 substitution/site” and have clarified its meaning in the figure legend:

“Branch lengths are proportional to the evolutionary distances inferred by IQ-TREE. The scale bar represents an evolutionary distance of one amino acid substitution per site.” (p. 22 line 767-769 in the revised manuscript)

**Reviewer #2 (Recommendations for the authors):**

(1) P. 9, in the "Data Accessibility Statement", all three PDB codes (23UX, 23UY, and 23UZ) should be listed.

We thank the reviewer for this comment. We carefully rechecked the Data Accessibility Statement and confirmed that all three PDB accession codes (23UX, 23UY, and 23UZ) are included in the statement.

(2) Supplementary Figures S2 and S3. The authors have written "Asn33" and "Asn52", instead of "Glu32" and "Asp51" in both the figure and figure legend of Supplementary Figure S3. They have also written "TND" instead of "NTD" in the figure legend.

“Asn33” and “Asn52” in Supplementary Fig. S3 are not typographical errors. Asn33 in aqMutL and Asn52 in aqGyrB are the residues that directly coordinate the Mg^2+^ ion and are distinct from the acidic residues discussed in this paper. To avoid confusion, we have added the following sentence to the legend of Supplementary Fig. S3:

“These Mg^2+^-coordinating asparagine residues are adjacent to, but distinct from, the second acidic residues Glu32 in aqMutL and Asp51 in aqGyrB examined in this study.” (p. 21 line 749-751 in the revised manuscript)

We have also corrected the typographical error “TND” to “NTD” in the figure legend. (p. 21 line 749 in the revised manuscript)
